# Deep Reinforcement Learning-Based Accurate Control of Planetary Soft Landing

**DOI:** 10.3390/s21238161

**Published:** 2021-12-06

**Authors:** Xibao Xu, Yushen Chen, Chengchao Bai

**Affiliations:** 1 School of Astronautics, Harbin Institute of Technology, Harbin 150001, China; xuxibaogz@163.com (X.X.); 20S018108@stu.hit.edu.cn (Y.C.); 2 Beijing Institute of Astronautical Systems Engineering, Beijing 100076, China

**Keywords:** soft landing, velocity tracking, deep reinforcement learning (DRL)

## Abstract

Planetary soft landing has been studied extensively due to its promising application prospects. In this paper, a soft landing control algorithm based on deep reinforcement learning (DRL) with good convergence property is proposed. First, the soft landing problem of the powered descent phase is formulated and the theoretical basis of Reinforcement Learning (RL) used in this paper is introduced. Second, to make it easier to converge, a reward function is designed to include process rewards like velocity tracking reward, solving the problem of sparse reward. Then, by including the fuel consumption penalty and constraints violation penalty, the lander can learn to achieve velocity tracking goal while saving fuel and keeping attitude angle within safe ranges. Then, simulations of training are carried out under the frameworks of Deep deterministic policy gradient (DDPG), Twin Delayed DDPG (TD3), and Soft Actor Critic (SAC), respectively, which are of the classical RL frameworks, and all converged. Finally, the trained policy is deployed into velocity tracking and soft landing experiments, results of which demonstrate the validity of the algorithm proposed.

## 1. Introduction

With the development of space technology, the scope of space exploration is constantly expanding. To further explore and study planets such as the Moon and Mars, a large number of planetary surface exploration missions have been carried out and many are in planning [1,2]. In planetary surface exploration missions, the lander faces many challenges. On the one hand, to avoid damaging onboard equipments, the landing velocity relative to the planet’s surface must be kept under a threshold. In addition, the maneuvering ability of the landing probe is limited, so the lander needs to have a high landing accuracy to explore a specific area. Therefore, precise soft landing guidance technology is always one of the key technologies of planetary exploration, which has been widely studied and achieved many achievements [3,4,5].

At present, soft landing algorithms can be roughly divided into five categories: establishing lunar vertical line, gravity turn guidance, nominal trajectory guidance, explicit guidance, and learning-based method.

The establishment of lunar vertical line is an open-loop guidance method, which requires high accuracy of orbit entry and mid-course correction [6]. Gravity turn guidance is a semi-open loop and semi-closed loop guidance method. In the main braking period, the main goal is to reduce the speed of the lander. While the distance between the lander and the lunar surface is shortened to a certain range, the closed-loop guidance works are based on the feedback information of the sensor to improve the landing accuracy and stability [7]. These two methods were generally only used in early lunar landings [8]. Nominal trajectory guidance consists of open-loop offline trajectory planning and closed-loop online trajectory tracking. Before landing, the lander needs to plan an optimized landing trajectory. During the landing process, the deviation between the lander position and velocity and the planned trajectory is constantly measured and eliminated, through which the lander is controlled to land at the desired landing sites [9,10,11,12]. Explicit guidance method solves the closed-loop guidance problem based on the explicit function of control functional. To obtain the analytical expression of the optimal control problem of soft landing, it is necessary to simplify the soft landing model when building the mathematical model [13]. The work in [14] proposes a guidance method for the powered soft landing of a launcher with non-cluster configured engines, for which it is difficult to maintain a low thrust-to-weight ratio. The work in [15] deals with designing soft landing trajectory, from lunar parking orbit to the surface of Moon and solve the optimization problem with Differential Evolution (DE) which is superior in convergence speed.

In recent years, machine learning has made great breakthroughs and development. Some researchers have studied soft landing based on intelligent learning algorithms such as deep learning. In [16], the deep architectures’ ability to drive the onboard decision-making system is investigated in detail. Under the assumption of perfect state information, deep networks are trained to approximate the optimal control action in pinpoint landing experiments and have the ability to cope with large sets of possible initial states. The work in [17] proposes an autonomous lunar landing method based on deep learning that takes raw images taken by onboard optimal cameras as input and directly outputs fuel-optimal control actions, in which the direct filters for state estimation is not necessary. Moreover, the deep networks are trained by a supervised machine learning algorithm, and the training datasets are generated by NLP solver software packages. Further, in [18], a recurrent neural network architecture is proposed to predict the fuel-optimal thrust from a sequence of states in the powered descent phase of planetary soft landing.

In future exploration missions, it is necessary to enable the lander with higher autonomy that can make real-time adjustments to landing trajectory according to the landing condition, which is difficult for offline planning methods.

As an important branch of machine learning, DRL integrates the perception ability of deep learning with the decision-making ability of RL. It is an end-to-end algorithm that directly takes the environmental information as inputs and outputs the control efforts. It is especially suitable for solving the decision-making and planning problems of complex systems and has been widely studied and applied in the fields of games [19], autonomous driving [20], and manipulator control [21].

In this paper, the problem of planetary soft landing of the powered descent phase is studied. The soft landing problem of the powered descent phase is formulated, and the theoretical basis of RL used in this paper is introduced. To make it easier for the training process to converge, a reward function including process reward is designed to solving the problem of sparse reward. In addition, the fuel consumption penalty and constraints violation penalty are included to save fuel and keep the attitude angle within constraints. The main contributions of this work are (i) a velocity tracking reward function is designed with process reward, which makes it easier for the lander to learn to achieve the goal of soft landing as well as enables it with a better generalization capability, and (ii) the goals of keeping the attitude within constraints and reducing fuel consumption are reached by including fuel consumption penalty and constraints violation penalty into the reward function.

The remainder of this paper is organized as follows. Section 2 gives the preliminaries about RL and formulates the soft landing as an RL problem. Section 3 describes the details about soft landing control method based on DRL. Simulation results and necessary discussions are given in Section 4. Finally, conclusions are presented in Section 5.

## 2. Preliminaries and Problem Formulation

### 2.1. Soft Landing Problem Formulation

**Figure 1:**
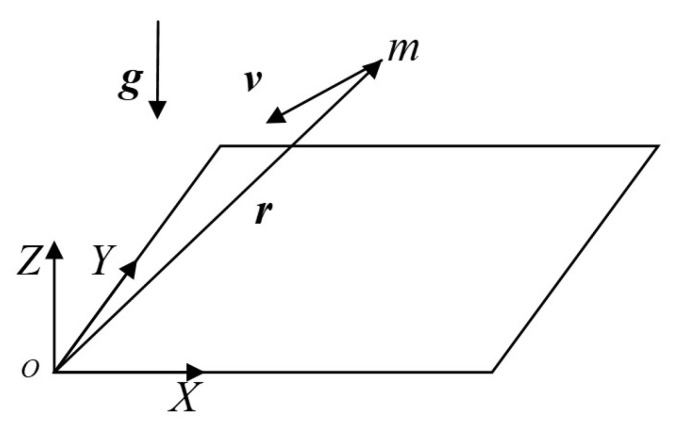
Navigation coordinates of planetary surface.

The planetary surface fixed frame of reference is defined as Figure 1. The forces acting on the lander in power descent include gravity, aerodynamic force, and engine thrust [22].

As the powered descent begins at an altitude that is quite low compared to the planet’s radius, and the distance between the lander and target landing sites varies slightly during this phase, it is appropriate to assume that the planet’s gravity is a constant g.

When it comes to the power descent phase, the lander has already released the parachute and the speed is on the order of 100 meters per second [22]. Compared with planetary gravity, the acceleration caused by aerodynamic force was very small. Therefore, the external force on the lander was dominated by gravity, and the aerodynamic force caused by the wind field was added into the model as an environmental disturbance.

**Figure 2:**
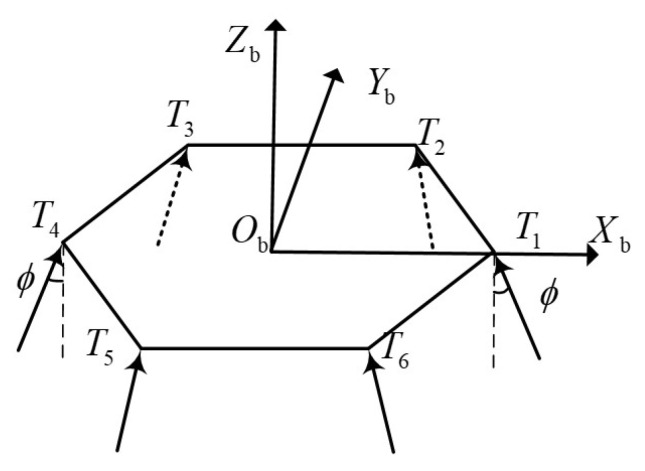
Lander body frame and thrusters layout. The thrusters are deployed in regular hexagon.

The lander is equipped with six thrusters deployed in body frame $O_{b}X_{b}Y_{b}Z_{b}$ as Figure 2.

The thrust of each engine is $T_{i}$, and meets constraint

(1) $$T_{\min}\leq T_{i}\leq T_{\max}$$

Let $T={\left[\begin{array}{cccccc} T_{1} & T_{2} & T_{3} & T_{4} & T_{5} & T_{6} \end{array}\right]}^{T}$ be the vector composed of the thrusts of six thrusters, and the thrust vector in body frame can be obtained according to the geometric relation

(2) $$T_{b}=\left[\begin{array}{cccccc} -\sin\phi & -\frac{1}{2}\sin\phi & \frac{1}{2}\sin\phi & \sin\phi & \frac{1}{2}\sin\phi & -\frac{1}{2}\sin\phi \\ 0 & -\frac{\sqrt{3}}{2}\sin\phi & -\frac{\sqrt{3}}{2}\sin\phi & 0 & \frac{\sqrt{3}}{2}\sin\phi & \frac{\sqrt{3}}{2}\sin\phi \\ -\cos\phi & -\cos\phi & -\cos\phi & -\cos\phi & -\cos\phi & -\cos\phi \end{array}\right]T$$

Moreover, the same for the torque vector in body frame

(3) $$M_{b}=L\left[\begin{array}{cccccc} 0 & -\frac{\sqrt{3}}{2}\cos\phi & -\frac{\sqrt{3}}{2}\cos\phi & 0 & \frac{\sqrt{3}}{2}\cos\phi & \frac{\sqrt{3}}{2}\cos\phi \\ \cos\phi & \frac{1}{2}\cos\phi & -\frac{1}{2}\cos\phi & -\cos\phi & -\frac{1}{2}\cos\phi & \frac{1}{2}\cos\phi \\ 0 & 0 & 0 & 0 & 0 & 0 \end{array}\right]T$$

The translation dynamics are expressed as

(4) $$\begin{array}{c} {\dot{v}}_{b}=\frac{T_{b}+C_{e}^{b}g}{m}+v_{b}\times \omega^{t}\\ \dot{r}=C_{b}^{e}v_{b} \end{array}$$

where $r={\left[\begin{array}{ccc} x & y & z \end{array}\right]}^{T}$ is the translational vector of the lander, v is the velocity vector, and $v_{b}={\left[\begin{array}{ccc} u & v & w \end{array}\right]}^{T}$ and $\omega^{t}=\left[\begin{array}{ccc} p & q & r \end{array}\right]$ are the velocity and angular velocity in the body frame, respectively. $Q=\left[\begin{array}{cccc} q_{0} & q_{1} & q_{2} & q_{3} \end{array}\right]$ is the quaternion. I and *m* are the inertia matrix and mass of the lander, respectively. $C_{b}^{e}$ is the direction cosine matrix from the body frame to the surface fixed frame of reference.

(5) $$\begin{array}{c} C_{b}^{e}={\left(C_{e}^{b}\right)}^{T}\\ C_{e}^{b}=C_{x}\left(\varphi \right)C_{y}\left(\theta \right)C_{z}\left(\psi \right) \end{array}$$

Attitude dynamics are express as

(6) $${\dot{\omega }}^{t}=I^{-1}\cdot \omega^{t}\times \left(I\cdot \omega^{t}\right)$$

(7) $$\begin{array}{c} \dot{\varphi }=p+\tan\theta \left(q\sin\varphi +r\cos\varphi \right)\\ \dot{\theta }=q\cos\varphi -r\sin\varphi \\ \dot{\psi }=\left(q\sin\varphi +r\cos\varphi \right)/\cos\theta \end{array}$$

During soft landing, the mass of the lander will gradually decrease with the fuel consumption, i.e.,

(8) $$\dot{m}=-\frac{1}{I_{\mathrm{sp}}}\sum_{i}T_{i}\left(t\right)$$

where $I_{\mathrm{sp}}$ is the specific impulse of the engine, and the inertia matrix will also gradually decrease as the mass decreases. The shape of the lander is a cuboid of sides of length a×b×c with uniform mass distribution

(9) $$\begin{array}{c} I=\left[\begin{array}{ccc} I_{xx} & 0 & 0\\ 0 & I_{yy} & 0\\ 0 & 0 & I_{zz} \end{array}\right]\\ I_{xx}=\frac{m}{12}\left(b^{2}+c^{2}\right)\\ I_{yy}=\frac{m}{12}\left(a^{2}+c^{2}\right)\\ I_{zz}=\frac{m}{12}\left(a^{2}+b^{2}\right) \end{array}$$

The soft landing problem is described as a fuel optimization problem as

(10) $$\min_{T_{i}\left(t\right)}J=\int_{t_{0}}^{t_{f}}\frac{1}{I_{\mathrm{sp}}}\sum_{i}T_{i}\left(t\right)dt$$

### 2.2. RL Basis

**Figure 3:**
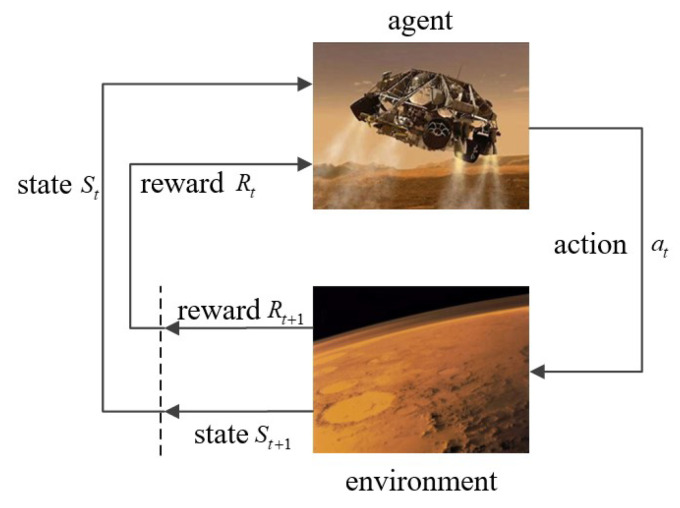
Interaction between agent and environment.

RL is a data-driven algorithm, which is different from supervised learning. RL obtains training data(experience) through interaction with the environment, as shown in Figure 3.

At time step *t*, the agent gets an observation $S_{t}=s$ from the environment, then takes an action $A_{t}=a$ according to the policy $\pi (A_{t}=a_{t}|S_{t}=s)$, the environment transfers to the next state $s^{\prime }$ based on the model $P(S_{t+1}=s^{\prime }|S_{t}=s,A_{t}=a_{t})$ and returns a reward $R_{t+1}$. Define the accumulated return of an episode as

(11) $$G_{t}=R_{t+1}+\gamma R_{t+2}+\gamma^{2}R_{t+3}+\ldots =\sum_{k=1}\gamma^{k-1}R_{t+k}$$

where $\gamma \in \left[0,1\right]$ is the discount factor. When γ→0, the agent only cares about the most recent rewards. While γ→1, the agent has a longer horizon and cares more about the future reward. Moreover, the target is to update policy to maximize $G_{t}$.

DDPG, TD3, and SAC are three of the most successful and popular RL algorithms, so we chose them as the framework in this paper.

(1) DDPG

DDPG is a deterministic policy RL framework that outputs a deterministic action, and it is the result of the deep Q network (DQN) extended to continuous control space. DDPG has been extensively researched and applied to the field of continuous control. It learns both a value function and a policy. First, it approximates the value function via the Bellman equation with offline experience through gradient descent. Then, the policy is updated by maximizing the approximated value function.

DDPG is one of the standard algorithms that training a deep neural network to approximate Q function. It makes use of the past experience through the trick of replay buffer. When it is time to update, it randomly samples from the buffer. In order to stabilize the training process, the size of the replay buffer should be properly chosen. If it is too small, it can only store recent experiences, which makes the policy brittle. However, if it is too large, the possibility of sampling a good experience will decrease, and it takes more episodes for the training process to converge.

As shown in Algorithm 1, the whole learning process consists of two parts: Q-learning and policy learning.

According to Bellman equation, optimal *Q* function under optimal policy satisfies

(12) $$Q^{*}\left(s,a\right)=\underset{s^{\prime }∼P}{E}\left[r\left(s,a\right)+\gamma \max_{a^{\prime }}Q^{*}\left(s^{\prime },a^{\prime }\right)\right]$$

where *P* is the environment model. Given the experience $\left(s,a,r,s^{\prime },d\right)$, value function $Q_{\pi_{\theta }}$ under the policy $\pi_{\theta }$ can be represented as

(13) $$Q_{\pi_{\theta }}\left(s,a\right)=\underset{\left(s,a,r,s^{\prime },d\right)∼D}{E}\left[r\left(s,a\right)+\gamma \max_{a^{\prime }}Q_{\pi_{\theta }}\left(s^{\prime },a^{\prime }\right)\right]$$

**Algorithm 1:** DDPG based soft landing.

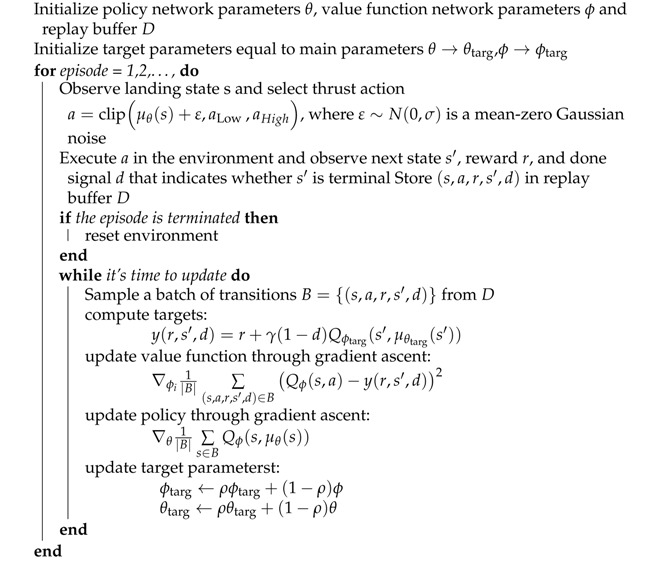



Setup a mean-squared Bellman error function as

(14) $$L\left(\phi ,D\right)=\underset{(s,a,r,s!d)∼D}{E}\left[{\left(Q_{\phi }\left(s,a\right)-\left(r+\gamma \left(1-d\right)\max_{a^{\prime }}Q_{\phi }\left(s^{\prime },a^{\prime }\right)\right)\right)}^{2}\right]$$

where $r+\gamma \left(1-d\right)\max_{a^{\prime }}Q_{\phi }\left(s^{\prime },a^{\prime }\right)$ is the value function target. In the condition of continuous action space, it is difficult to compute action $a^{\prime }$ which maximizes $Q_{\phi }\left(s^{\prime },a^{\prime }\right)$. Therefore, DDPG uses target network to solve this problem

(15) $$\max_{a^{\prime }}Q_{\phi }\left(s^{\prime },a^{\prime }\right)=Q_{\phi_{\mathrm{targ}}}\left(s^{\prime },\mu_{\theta_{\mathrm{targ}}}\left(s^{\prime }\right)\right)$$

where $a^{\prime }$ is obtained via target policy $\mu_{\theta_{\mathrm{targ}}}$. With the training process going on, policy and value function will gradually converge to optimal policy and optimal value function respectively.

(2) TD3

TD3 is modified from DDPG. DDPG can perform well sometimes but it highly depends on the choice of hyperparameters. TD3 takes three critical tricks to make the training process more stable.

- ***Clipped Double-Q Learning.*** TD3 learns two value function networks at the same time. When calculating the target, and are input to the two target value function networks at the same time after obtaining. When the value function network is updated, the smaller one is selected to compute the loss function of the error of the Bellman equation.
  (16) $$y\left(r,s^{\prime },d\right)=r+\gamma \left(1-d\right)\min_{i=1,2}Q_{\phi_{t\arg,i}}\left(s^{\prime },{\tilde{a}}^{\prime }\left(s\right)\right)$$
- ***Target Policy Smoothing.*** The value function learning method of TD3 and DDPG is the same. When the value function network is updated, noise is added to the action output of the target policy network to avoid overexploitation of the value function
  (17) $$\max_{a^{\prime }}Q_{\phi }\left(s^{\prime },a^{\prime }\right)=Q_{\phi_{\mathrm{targ}}}\left(s^{\prime },\pi_{\theta_{\mathrm{targ}}}\left(s^{\prime }\right)+\varepsilon \right)$$
  where ε∼N(0,σ) is a mean-zero Gaussian noise, and $\theta_{\mathrm{targ}}$ is the parameters of the target strategy network. Adding noise to the output action of the strategy network serves as regularization, which avoids the overexploitation of value function and stabilizes the training process.
- ***Delayed Policy Updates.*** As the output of the target strategy network is used to compute the target of the value function, the agent can be brittle because of frequent strategy updates, so TD3 adopts the Delayed Policy Updates trick. When updating the strategy network, the update frequency of the strategy network is lower than that of the value function network. This helps to suppress the training fluctuation and makes the learning process more stable.

(3) SAC

SAC is an RL framework that maximizes cross-entropy. It applies the learning techniques of DDPG to the learning of random strategies and optimizes random strategies in an offline learning mode.

The agent starts from the initial state $s_{0}$∼$p(s_{0})$, samples from policy distribution $a_{t}$∼$\pi \left(\cdot |s_{t}\right)$, and gets an action $a_{t}$ acting on the environment. Then, the environment returns a reward $r(s_{t},a_{t})$ and transfers to a new state $s_{t+1}$∼$p\left(\cdot |s_{t},a_{t}\right)$ according to the environmental model. Repeating the interacting process and the trajectory of the state, $\tau =(s_{0},a_{0},s_{1},a_{1},\ldots )$ can be obtained. The probability distribution of the trajectory τ regarding the strategy π is expressed as

(18) $$\rho_{\pi }\left(\tau \right)=p\left(s_{0}\right)\prod_{t}\pi \left(a_{t}|s_{t}\right)p\left(s_{t+1}|s_{t},a_{t}\right)$$

The maximum cross-entropy RL optimizes the cumulative return and cross-entropy of the strategy. For Markov decision processes (MDPs) with infinite loss rewards, the optimization objective can be expressed as

(19) $$J\left(\pi \right)=\sum_{t=0}^{\infty }E_{\tau ∼\rho_{x}}\left[\sum_{t=0}^{\infty }\gamma^{t}\left[r\left(s_{t},a_{t}\right)-\alpha \log\pi \left(a_{t}|s_{t}\right)\right]\right]$$

Moreover, the optimal policy is represented as

(20) $$\pi^{*}=\arg\max_{\pi }E_{\tau ∼\rho_{x}}\left[\sum_{t=0}^{\infty }\gamma^{t}\left[r\left(s_{t},a_{t}\right)-\alpha \log\pi \left(a_{t}|s_{t}\right)\right]\right]$$

where $\log\pi_{t}\left(a_{t}|s_{t}\right)$ is the cross-entropy of strategy distribution, which can be added into the optimization target to encourage agents to explore the environment in training, and to improve the robustness of training results. α is the temperature coefficient, which is used to adjust the importance of cross-entropy, and thus plays a role in regulating the randomness of the optimal strategy. A large α encourages the agent to explore the environment. Therefore, the larger the α is, the more stochastic the strategy will be. While the smaller alpha is, it is more likely that the policy falls into a local optimal point. When α→0, maximizing cross-entropy RL degenerates into conventional RL that maximizes cumulative reward.

Based on the optimization objective above, the state value function is defined as

(21) $$V^{\pi }\left(s\right)=E_{\tau ∼\rho_{x}}\left[\sum_{t=0}^{\infty }\gamma^{t}\left[r\left(s_{t},a_{t}\right)-\alpha \log\pi \left(\cdot |s_{t}\right)\right]|s_{0}=s\right]$$

Moreover, the value function

(22) Qπ(s,a)=Eτ∼ρπs0=s,a0=a∑t=0∞γtrst,at−α∑t=1∞γtlogπ·∣st

(23) $$\begin{array}{c} \;V^{\pi }\left(s\right)=E_{a∼\pi }\left[Q^{\pi }\left(s,a\right)\right]-\alpha \log\pi \left(\cdot |s\right) \end{array}$$

Then, according to the Bellman equation,

(24) Qπ(s,a)=Es′∼pr(s,a)+γVπs′=Es′∼pa′∼πr(s,a)+γQπs′,a′−αlogπ·∣s′

SAC makes use of “Clipped Double Q-learning” and the Q-learning is similar to TD3 except for the compute of the target value function

(25) $$y\left(r,s^{\prime },d\right)=r+\left(1-d\right)\left(\min_{j=1,2}Q_{\phi_{u}\mathrm{eg},j}\left(s^{\prime },a^{\prime }\right)-\alpha \log\pi_{\theta }\left(a^{\prime }|s^{\prime }\right)\right)$$

According to Equation (24), then

(26) $$\begin{array}{c} \max_{\theta }V^{\pi_{\theta }}\left(s\right)=\max_{\theta }\underset{a∼\pi_{\theta }}{E}\left[Q^{\pi_{\theta }}\left(s,a\right)-\alpha \log\left(\pi_{\theta }\left(a|s\right)\right)\right]\\ =\max_{\theta }\underset{\xi ∼N}{E}\left[Q^{\pi_{\theta }}\left(s,{\tilde{a}}_{\theta }\left(s,\xi \right)\right)-\alpha \log\left(\pi_{\theta }\left({\tilde{a}}_{\theta }\left(s,\xi \right)|s\right)\right)\right] \end{array}$$

The updating of the policy network makes use of the re-parameterization trick

(27) $$a={\tilde{a}}_{\theta }\left(s,\xi \right)=\tanh\left(\mu_{\theta }\left(s\right)+\sigma_{\theta }\left(s\right)⊙\xi \right)\;\xi ∼N\left(0,1\right)$$

where the action distribution is Gaussian; $\mu_{\theta }\left(s\right)$ and $\sigma_{\theta }\left(s\right)$ are the mean value and variance of the Gaussian distribution, respectively; and ξ is the standard Gaussian distribution. After sampling from the distribution, the action output is restricted to the constrained range through the Tanh activation function.

Besides, SAC makes use of the Clipped *Q* trick when updating its strategy

(28) $$Q^{\pi_{\theta }}\left(s,a\right)=\min_{j=1,2}Q_{\phi_{j}}\left(s,{\tilde{a}}_{\theta }\left(s,\xi \right)\right)$$

The strategy optimization objective is finally represented as

(29) $$\max_{\theta }\underset{s∼D}{E}\left[\min_{j∼∼}Q_{\phi_{j}}\left(s,{\tilde{a}}_{\theta }\left(s,\xi \right)\right)-\alpha \log\left(\pi_{\theta }\left({\tilde{a}}_{\theta }\left(s,\xi \right)|s\right)\right)\right]$$

Because of the inherent stochasticity, SAC can effectively avoid the overexploitation of value function.

## 3. Soft Landing with DRL

Based on the dynamic model established above, in this section we will design an algorithm based on RL according to the characteristics of soft landing problems, including the selection of observation values and the design of reward function and other settings concerning how the agent interacts the environment.

### 3.1. Reward Setting

The reward function is an index to evaluate the behavior of agents, which is directly related to the training result.

- ***Goal achieving reward:*** When the altitude of the lander is less than 0, the speed is downward, the speed is less than the upper limit of soft landing speed, and the attitude angle and angular rate is within the limited range, the lander is considered to have achieved soft landing, and gets the reward
  (30) $$\begin{array}{c} r_{goal}=\lambda (h<0\;\mathrm{and}\;v_{z}<0\;\mathrm{and}\;∥v∥<v_{\lim}\;\mathrm{and}\\ \;\;\phi <\phi_{\lim}\;\mathrm{and}\;\theta <\theta_{\lim}\;\mathrm{and}\;\psi <\psi_{\lim}\;\mathrm{and}\\ \;\;∥\omega ∥<\omega_{\lim}) \end{array}$$
  where λ is a large positive constant serving as a soft landing bonus. $\phi_{\lim},\theta_{\lim},\psi_{\lim}$ are upper bounds of Euler angle of the lander, and $\omega_{\lim}$ is the upper bounds of angular rate.
- ***Velocity tracking reward:*** At the beginning of the phase of powered descent, the lander is several kilometers away from the landing zone, and the initial velocity is around 100 m/s. If the agent is rewarded only when it achieves a soft landing at the target area, the state space is so sparse that it’s nearly impossible to converge. Therefore, we transfer the soft landing problem into a velocity tracking problem. The process reward is introduced in the landing process, that is, a reference velocity is given according to the real-time relative position between the lander and target landing area
  (31) $$v_{ref}=\left\{\begin{array}{cc} -\frac{r-r_{1}}{k_{v1}} & h\geq h_{1}\\ -\frac{r-r_{2}}{k_{v2}} & 0\leq h<h_{1} \end{array}\right.$$
  where $k_{v1}$ and $k_{v2}$ are constant coefficients that determine the mapping relationship between position and reference velocity, and large coefficients lead to smaller reference velocity in the powered descent process. Moreover, the reward is given according to the deviation between the real velocity and the reference velocity of the lander
  (32) $$r_{vel}=\beta ∥v-v_{ref}∥$$
  where β is the reward coefficient of velocity tracking error.
- ***Crash penalty:*** To avoid the crash of the lander, a penalty is included in the reward. When the attitude angle or speed deviation exceeds the threshold, the episode terminates and the environment returns a large negative reward as a penalty
  (33) $$r_{crash}=\eta \left(\phi >\phi_{\lim}\;or\;\theta >\theta_{\lim}\;or\;\psi >\psi_{\lim}\right)$$
  where η is the penalty of attitude crash.
- Fuelconsumptionpenalty: In planetary exploration missions, the fuel carried by the lander is limited, so the fuel consumption should be minimized. A reward regarding fuel consumption is defined as
  (34) $$r_{fuel}=\alpha \frac{1}{I_{sp}}\sum_{i=1}^{6}T_{i}$$
  where α weights a term penalizing fuel consumption. The fuel consumption coefficient α and velocity tracking error coefficient β explicitly control the trade-off between fuel consumption and velocity tracking. With higher |α| and lower |β|, fuel consumption weights more in the reward and the lander will exchange some velocity tracking performance for less fuel consumption.
- ***Constant reward:*** Notice that the rewards $r_{vel}$, $r_{crash}$, $r_{fuel}$ are all negative. To encourage the agent to explore more, a positive constant reward needs to be introduced into the reward.
  (35) $$r_{constant}=\kappa$$

Therefore, the overall reward format is as follows:

(36) $$r=r_{fuel}+r_{vel}+r_{crash}+r_{constant}+r_{goal}$$

### 3.2. Observation Space

To improve the landing performance, it is necessary to include position, velocity, attitude, and angular rate into the observation $s_{t}$. Based on the analysis of reward setting, the absolute velocity in $s_{t}$ is replaced by the velocity deviation in the lander body frame.

(37) $$\delta v_{b}=C_{e}^{b}\left(v-v_{ref}\right)$$

Tracking velocity deviation rather than direct position can improve the generalization ability of the trained agent.

Each attitude Angle is input into the observation vector in the form of sine value and cosine value, and the quaternion is observed simultaneously.

(38) $$\begin{array}{c} s=[\delta v_{\mathrm{bx}},\delta v_{\mathrm{by}},\delta v_{\mathrm{bz}},\sin\left(\phi \right),\cos\left(\phi \right),\sin\left(\theta \right),\cos\left(\theta \right),\\ \;\sin\left(\psi \right),\cos\left(\psi \right),p,q,r,q_{0},q_{1},q_{2},q_{3}] \end{array}$$

### 3.3. Action Space

The lander’s action is the thrusts of the engines, which are bounded in a specific range. After going through the Tanh activation function, the output of the policy network is bounded to $a_{i}\in \left[-1,1\right]$. Then, the actual thrust is got through a linear mapping

(39) $$T_{i}=\frac{T_{\max}-T_{\min}}{2}a_{i}+\frac{T_{\max}+T_{\min}}{2}$$

where $T_{\min}$ and $T_{\max}$ are the lower and upper bounds of the thrust, respectively.

### 3.4. Network Architecture

We use the deep learning framework PyTorch to build the neural networks. The hyperparameters such as network learning rates and noise variance are defined Section 4.

**Figure 4:**
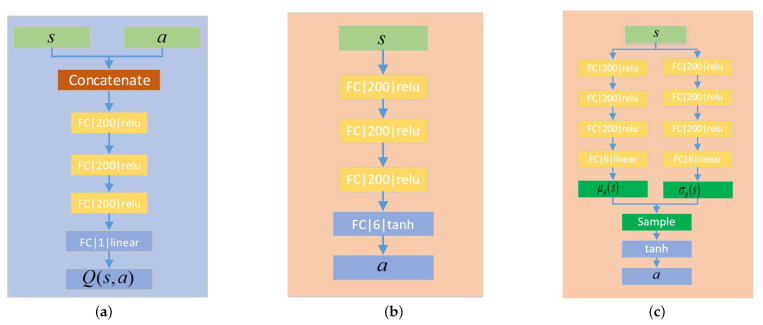
Network architecture. (**a**) Value function network. (**b**) Policy network of DDPG and TD3. (**c**) Policy network of SAC.

DDPG, TD3, and SAC all contain value function networks and policy networks. In this paper, all the networks of value functions have the same structure as Figure 4a. We employ three hidden layers to process the vector concatenated of observation and action. All the hidden layers contain 200 nonlinear units and the activation function is ReLU.

The policy network structure of DDPG and TD3 is the same, as shown in Figure 4b. The network includes three hidden layers, of which the activation function is ReLU, and the activation function of the output layer is Tanh, through which the action is normalized to [−1, 1].

Different from the deterministic policy of DDPG and TD3, SAC is a stochastic strategy and the structure of the policy network is shown in Figure 4c. The strategy network consists of two paths of networks. Both of them have the same structure, which outputs the mean value and variance of the Gaussian distribution, respectively. Then, the network output is obtained by sampling and activation functions in turn.

## 4. Simulation Results and Discussion

In this section, the simulation experiments are carried out and the results and related discussions are proposed.

### 4.1. Simulation Settings

We train the lander velocity controller in a 6DOF environment established in Section 2. The environmental parameters settings are shown in Table 1.

The hyper parameter settings of DDPG, TD3, and SAC algorithms are shown in Table 2, Table 3 and Table 4, respectively. The training of DDPG is unstable and the learning rate of its value function is lower than TD3.

### 4.2. Simulation Results

**Figure 5:**
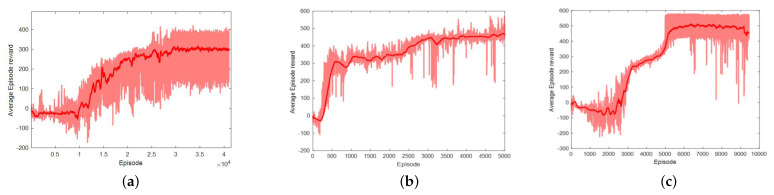
The curves of accumulative reward for each training episode. (**a**) DDPG. (**b**) TD3. (**c**) SAC.

We deployed training algorithms according to the setting of environment and algorithm parameters listed above, and the reward change curves of DDPG, TD3, and SAC in the training process are shown in Figure 5.

The dark red curve is the average reward. From Figure 5a, the episode reward starts to rise at around episode 10,000 and continues to increase until episode 20,000. Though the curve of average reward looks stable, the episode reward fluctuates between 100 and 400, which is very unstable.

It is obvious from the reward curve that the performance of the TD3 agent improves significantly after 700 episodes of training. After 5000 episodes of training, the average reward converges at 450. Compared with DDPG, the learning speed of TD3 is faster and the performance is more stable, which is the result of the three tricks of “Clipped Double-Q Learning”, “Target Policy Smoothing”, and “Delayed Policy Updates”.

The training of the SAC agent experienced a significant improvement at around episode 3000 and 5000, respectively, and finally stabilized and the reward converged to 500. Due to the introduction of the “Clipped Double-Q Learning” like TD3, plus the inherent smoothing characteristics of the stochastic strategy, the training process of SAC fluctuates within a very small range, and the exploration of the environment is sufficient. The accumulated rewards of some episodes are close to 600, which is higher than DDPG and SAC.

**Figure 6:**
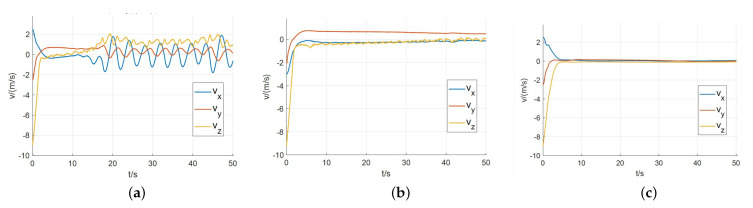
The curves of velocity deviation in velocity tracking experiments. (**a**) DDPG. (**b**) TD3. (**c**) SAC.

The reference velocity is set as $v_{d}=\left[\begin{array}{ccc} 0 & 0 & 0 \end{array}\right]m/s$, and the policy obtained from training is used to control the speed. With the initial velocity $v_{b}^{0}=\left[\begin{array}{ccc} -2.0 & 2.0 & 10.0 \end{array}\right]m/s$, the curves of the velocity deviation are shown in Figure 6.

All of the trained controllers can keep the velocity deviation within a certain range. But there is a continuous oscillation in the velocity control by DDPG agent. The agent trained by SAC algorithm performs the best, which gets the velocity error converges to 0 and keeps it stable.

Besides velocity tracking experiments, we validate the trained policy of each algorithm by soft landing tests. Through 100 shooting experiments, landing statistics are shown in Table 5. Among the three, SAC has the highest landing success rate of 96%, while DDPG terminates 26 times due to attitude over constraint, which is caused by continuous oscillation when tracking reference velocity. In the soft landing process, the reference velocity changes dynamically, causing the oscillation more serious and leading to a higher failure rate.

**Figure 7:**
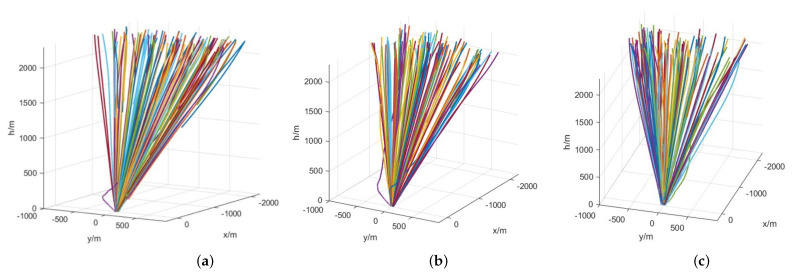
Landing trajectories of lander under the control of the trained velocity controller. (**a**) DDPG. (**b**) TD3. (**c**) SAC.

**Figure 8:**
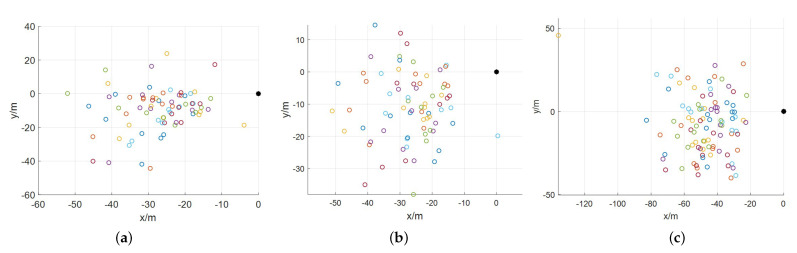
2D distribution of landing points. (**a**) DDPG. (**b**) TD3. (**c**) SAC.

Taking the target landing site as the origin, the landing trajectory and landing point distribution of the three controllers are shown in Figure 7 and Figure 8, respectively. By analyzing the distribution of landing points, DDPG and SAC have nearly the same accuracy of 100 m in successful landing cases, while some landing points of SAC distance 200 m from the origin.

## 5. Conclusions

This paper presents an end-to-end soft landing control algorithm based on RL. First, the 6DOF soft landing dynamics model is established and the soft landing problem of the powered descent phase is formulated. The theoretical basis of RL is briefly introduced. Then, to solve the problem of sparse reward, which makes it hard for the policy to converge, the reward function including process reward is designed. Besides, the fuel consumption penalty and constraints violation penalty are included in the reward function to optimize fuel consumption and keep attitude angle within constraints. Moreover, the networks architecture of the RL algorithms used is designed. The value functions of DDPG, TD3, and SAC are approximated by deep neural networks that have the same architecture. Both DDPG and TD3 have a policy network that outputs deterministic action, while the action output of SAC is sampled according to a Gaussian distribution characterized by the output of its policy network. Finally, simulations of training are carried out to evaluate the algorithm proposed. The results show that the performance varies between different RL frameworks, and the agent trained by SAC tracks the reference velocity best. In addition, the trained policy is deployed to soft landing experiments, results of which demonstrate the validity of the algorithm proposed. Future work will focus on the stability guarantee of RL-based soft landing algorithm, which is of great importance in space exploration missions to ensure success.

## Figures and Tables

**Table 1:** Parameter settings of environment.

Parameters	Values
$m_{0}$	1700 kg
a×b×c	3m×3m×1m
$I_{sp}$	225 s
$T_{max}$	2880 N
$T_{min}$	1080 N
*L*	2 m
ϕ	27°
$T_{s}$	0.2 s
$T_{f}$	50 s
$k_{v1}$	20
$k_{v2}$	20
$\delta v_{\mathrm{bx}0}$	[−3,3]m/s
$\delta v_{\mathrm{by}0}$	[−3,3]m/s
$\delta v_{\mathrm{bz}0}$	[−12,5]m/s

**Table 2:** Parameter settings of DDPG.

Parameters	Values
σ	0.02
$\left|D\right|$	$10^{6}$
ρ	0.998
Learning rate of policy networks	$10^{-3}$
Learning rate of value function networks	$5\times 10^{-4}$

**Table 3:** Parameter settings of TD3.

Parameters	Values
$\sigma_{1}$	0.02
$\sigma_{2}$	0.02
*c*	0.02
$\left|D\right|$	$10^{6}$
ρ	0.995
Learning rate of policy networks	$10^{-3}$
Learning rate of value function networks	$10^{-3}$

**Table 4:** Parameter settings of SAC.

Parameters	Values
$\left|D\right|$	$10^{6}$
ρ	0.995
α	0.05
Learning rate of policy networks	$10^{-3}$
Learning rate of value function networks	$10^{-3}$

**Table 5:** Landing success rate.

Parameters	DDPG	TD3	SAC
Number of experiments	100	100	100
Success rate of soft landing	74%	92%	96%

## Data Availability

Not applicable.

## Abbreviations

The following abbreviations are used in this manuscript:

DRL	Deep reinforcement learning
DDPG	Deep deterministic policy gradient
TD3	Twin Delayed DDPG
SAC	Soft Actor Critic
6DOF	6 Degree of Freedom
NLP	Nonlinear Programming
g	planet gravity
$T_{i}$	thrust of each engine
$T_{\min}$	minimum thrust of each engine
$T_{\max}$	maximum thrust of each engine
ϕ	cant angle for the engines
T	thrust vector composed of each engine
$T_{b}$	thrust vector in lander body frame
$M_{b}$	torque vector in lander body frame
r	surface relative lander position vector
$v_{b}$	velocity vector
$C_{b}^{e},C_{e}^{b}$	direction cosine matrix between
	lander body frame and planet surface frame
$\omega^{t}$	angular rate vector in lander body frame
Q	attitude quaterion
*m*	lander mass
I	inertia matrix of the lander
$I_{\mathrm{sp}}$	specific impulse for thrusters
a,b,c	lander sides of length
*J*	optimization target
$G_{t}$	agent accumulated return in an episode
$v_{\lim},\varphi_{\lim},\psi_{\lim},\theta_{\lim},\omega_{\lim}$	limited range for velocity, attitude angle and angular rate to
	achieve soft landing
$v_{ref}$	reference velocity
λ,α,β,η,κ	reward coefficients
$T_{s}$	simulation sample time
$T_{f}$	maximum simulation time in one episode
